# Key Role of the Scavenger Receptor MARCO in Mediating Adenovirus Infection and Subsequent Innate Responses of Macrophages

**DOI:** 10.1128/mBio.00670-17

**Published:** 2017-08-01

**Authors:** Mareike D. Maler, Peter J. Nielsen, Nicole Stichling, Idan Cohen, Zsolt Ruzsics, Connor Wood, Peggy Engelhard, Maarit Suomalainen, Ildiko Gyory, Michael Huber, Joachim Müller-Quernheim, Wolfgang W. A. Schamel, Siamon Gordon, Thilo Jakob, Stefan F. Martin, Willi Jahnen-Dechent, Urs F. Greber, Marina A. Freudenberg, György Fejer

**Affiliations:** a Max Planck Institute of Immunobiology and Epigenetics, Freiburg, Germany; b Allergy Research Group, Department of Dermatology, Medical Center-University of Freiburg, Faculty of Medicine, University of Freiburg, Freiburg, Germany; c Faculty of Biology, University of Freiburg, Freiburg, Germany; d Institute of Molecular Life Sciences, University of Zurich, Zurich, Switzerland; e Institute of Virology, Medical Center-University of Freiburg, Faculty of Medicine, University of Freiburg, Freiburg, Germany; f School of Biomedical and Healthcare Sciences, Peninsula Schools of Medicine and Dentistry, University of Plymouth, Plymouth, United Kingdom; g Department of Pneumology, Medical Center-University of Freiburg, Faculty of Medicine, University of Freiburg, Freiburg, Germany; h Department of Molecular and Cell Biology, University of Leicester, Leicester, United Kingdom; i Department of Dermatology and Allergology, University Medical Center, Justus Liebig University Giessen, Giessen, Germany; j BIOSS Centre for Biological Signalling Studies, Faculty of Biology and Center for Chronic Immunodeficiency CCI, Faculty of Medicine, Medical Center, University of Freiburg, Freiburg, Germany; k Sir William Dunn School of Pathology, University of Oxford, Oxford, United Kingdom; l Department of Dermatology and Allergology, University Medical Center Giessen and Marburg, Justus Liebig University Giessen, Giessen, Germany; m Biointerface Laboratory, Helmholtz Institute for Biomedical Engineering, Aachen, Germany; Brown University

**Keywords:** IL-1α, MARCO, MPI cells, adenovirus, cGAS, cytokines, innate immunity, macrophages, scavenger receptor

## Abstract

The scavenger receptor MARCO is expressed in several subsets of naive tissue-resident macrophages and has been shown to participate in the recognition of various bacterial pathogens. However, the role of MARCO in antiviral defense is largely unexplored. Here, we investigated whether MARCO might be involved in the innate sensing of infection with adenovirus and recombinant adenoviral vectors by macrophages, which elicit vigorous immune responses *in vivo*. Using cells derived from mice, we show that adenovirus infection is significantly more efficient in MARCO-positive alveolar macrophages (AMs) and in AM-like primary macrophage lines (Max Planck Institute cells) than in MARCO-negative bone marrow-derived macrophages. Using antibodies blocking ligand binding to MARCO, as well as gene-deficient and MARCO-transfected cells, we show that MARCO mediates the rapid adenovirus transduction of macrophages. By enhancing adenovirus infection, MARCO contributes to efficient innate virus recognition through the cytoplasmic DNA sensor cGAS. This leads to strong proinflammatory responses, including the production of interleukin-6 (IL-6), alpha/beta interferon, and mature IL-1α. These findings contribute to the understanding of viral pathogenesis in macrophages and may open new possibilities for the development of tools to influence the outcome of infection with adenovirus or adenovirus vectors.

## IMPORTANCE

Macrophages play crucial roles in inflammation and defense against infection. Several macrophage subtypes have been identified with differing abilities to respond to infection with both natural adenoviruses and recombinant adenoviral vectors. Adenoviruses are important respiratory pathogens that elicit vigorous innate responses *in vitro* and *in vivo*. The cell surface receptors mediating macrophage type-specific adenovirus sensing are largely unknown. The scavenger receptor MARCO is expressed on some subsets of naive tissue-resident macrophages, including lung alveolar macrophages. Its role in antiviral macrophage responses is largely unexplored. Here, we studied whether the differential expression of MARCO might contribute to the various susceptibilities of macrophage subtypes to adenovirus. We demonstrate that MARCO significantly enhances adenovirus infection and innate responses in macrophages. These results help to understand adenoviral pathogenesis and may open new possibilities to influence the outcome of infection with adenoviruses or adenovirus vectors.

## INTRODUCTION

Virus-macrophage interactions play crucial roles in the pathogenesis and control of viral infections. Since intracellular pathogen sensing is central in the elicitation of early antiviral responses, early interactions of viral pathogens with innate immune cells, including macrophages, contribute significantly to effective antiviral defense mechanisms (1, 2). Macrophages differ in their origin, development, and function (3). Since viruses use a range of surface molecules as receptors and facilitators for cellular entry (4), cell surface heterogeneity in macrophage subsets is expected to influence pathogen entry and also the innate stimulatory activity of different virus types.

Adenoviruses (Ads) are important, nonenveloped, double-stranded-DNA-containing pathogens. Replication-defective recombinant Ad vectors are used for vaccination against infectious diseases, for cancer immunotherapy, and for studies of virus-host cell interactions (5, 6). Human Ad species C members (e.g., Ad type 2 [Ad2] and Ad5) are important tools in such applications (7, 8). Ad infection elicits potent innate immune responses *in vitro* and *in vivo*, and mononuclear phagocytes are major contributors to these responses (9). Different populations of tissue macrophages and dendritic cells produce high levels of proinflammatory cytokines and type I interferons (IFNs) in response to Ad particles. These responses can critically influence the course of natural Ad infection or the outcome of Ad vector applications, for example, by tuning the expression of cell surface receptors on epithelial cells (10).

Virus entry triggers signaling pathways that play key roles in Ad-elicited proinflammatory responses, but the contributions of cellular factors that mediate Ad entry into resident tissue macrophages, such as surface receptors, are incompletely understood (11, 12). The coxsackievirus-Ad receptor (CAR) and integrins are the main surface receptors for Ad entry into epithelial cells. *In vitro* studies have shown that Ads attach to these cells by the interaction of the viral fiber knob with CAR, leading to the exposure of membrane-lytic protein VI (13). Viruses are subsequently internalized via receptor-mediated endocytosis, escape to the cytosol, and import viral DNA into the nucleus (12, 14–17). In liver cells *in vivo*, Ad particles can also be internalized via binding to cell membrane heparan sulfate proteoglycans through blood factors (18, 19).

While macrophages and other myeloid cells do not express CAR, interactions with other host factors have been shown to contribute to Ad entry into these cells. This includes opsonization by serum factors such as antibodies or complement, leading to viral entry into macrophages and neutrophils via ubiquitously expressed Fc and complement receptors (20, 21). Contributions to virus entry by serum lactoferrin and coagulation factor X have also been reported (19, 22).

Furthermore, the scavenger receptors (SRs) SR-A and SREC1, members of a family of pattern recognition receptors with broad ligand specificity that are expressed ubiquitously on macrophages, have been reported to play a role in Ad uptake into macrophages (21–25).

Several sensors have been implicated in the innate activation of mononuclear phagocytes by Ads. Toll-like receptor 2 (TLR2), TLR4, and TLR9 have been shown to contribute to Ad-induced cytokine production (26, 27). Efficient triggering of innate responses was shown to require endosomal escape and cytoplasmic detection of the virus (28). Recently, the cyclic GMP-AMP synthase (cGAS) has been identified as a major cytosolic innate sensor for Ads (29, 30).

Previously, we have demonstrated that the production of innate cytokines in response to respiratory pathogens, including Ads, is significantly stronger in murine AMs and in nontransformed, granulocyte-macrophage colony-stimulating factor-dependent, self-renewing, AM-like macrophages (designated Max Planck Institute [MPI] cells) than in bone marrow-derived macrophages (BMMs) (31). Similar to AMs, MPI cells express high levels of the SR class A protein MARCO, while rather Ad-insensitive BMMs do not express this SR. Functionally, MARCO has been shown to directly enhance antibacterial macrophage responses (32). Whether it plays a similar role in macrophage responses to viral infections has not yet been investigated.

In this study, we investigated the possibility that MARCO expression enhances the susceptibility of macrophages to Ad. Using several mouse macrophage types, we show that the presence of MARCO allows rapid Ad gene expression and strong virus-stimulated innate responses while blockage or a lack of MARCO results in strong impairment of these processes. Thus, MARCO is involved in innate Ad recognition and the elevated Ad sensitivity of MARCO-expressing macrophages is due to the expression of this receptor on the cell surface.

## RESULTS

### Ad entry into and activation of innate signaling in MPI cells and AMs, unlike those in BMMs, are fast and efficient.

**FIG 1:**
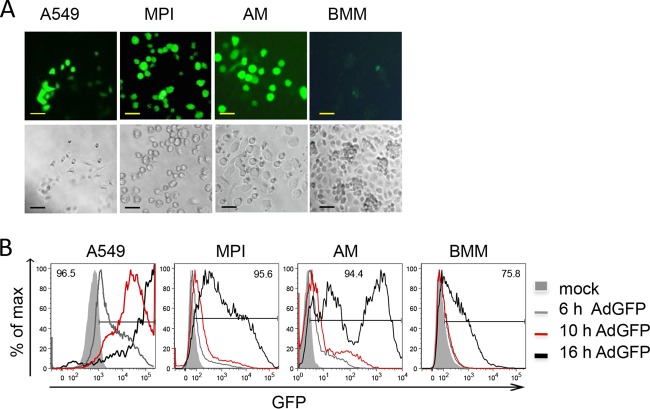
Efficiency of Ad infection in different cell types. (A) GFP expression was analyzed in different cell types 16 h p.i. with AdGFP by fluorescence (top) and light (phase-contrast; bottom) microscopy. Bars, 50 µm. (B) FACS analysis of GFP expression in infected cells at the time points indicated. The value at the top of each graph is the frequency of GFP^+^ cells at 16 h p.i., and the gate indicates GFP^+^ cells.

Intracellular sensing of incoming virus particles is a major process in the triggering of antiviral responses. We tested the efficiency of adenoviral transduction of various macrophage types, i.e., alveolar macrophages (AMs), BMMs, and MPI cells, by using a nonreplicating, green fluorescent protein (GFP)-expressing Ad. The lung epithelial cell line A549, which can be infected efficiently with Ads, was also used as a positive control. Microscopic examination at 16 h postinfection (p.i.) revealed that the majority of the MPI cells, AMs, and A549 cells were strongly GFP positive, while BMMs expressed GFP only weakly (Fig. 1A). The time course of GFP expression in infected cells was analyzed by flow cytometry, which showed that GFP expression was already detectable at 6 h p.i. in A549 and MPI cells and AMs, whereas BMMs were still GFP negative at 10 h p.i. (Fig. 1B). After 16 h, about 94 to 96% of the A549 and MPI cells and AMs expressed GFP. In MPI cells and AMs, two populations, moderately and strongly positive subsets, could be identified. In contrast, only 75% of the BMMs were weakly GFP positive by this late time point. These results demonstrated faster and more efficient transduction of MPI cells and AMs than of BMMs.

**FIG 2:**
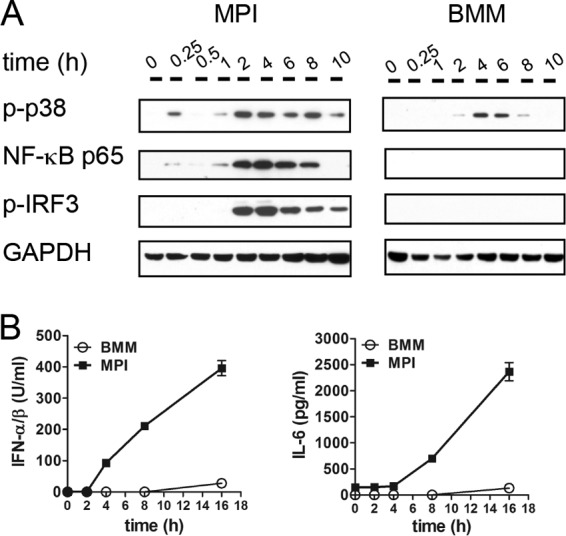
Early immune activation of MPI cells and BMMs p.i. with AdGFP. (A) Western blot analysis of the cytoplasmic (p-p38, p-IRF3) and nuclear (NF-κB p65) cell fractions at the time points indicated. (B) Cytokine production was measured by ELISA in cell-free supernatants at the time points indicated. GAPDH, glyceraldehyde-3-phosphate dehydrogenase.

To compare the kinetics and strength of the Ad-induced signaling and cytokine responses in the different macrophage types, we measured the activation of the transcription factors IRF3 and NF-κB, the activation of the mitogen-activated protein kinase p38, and the secretion of IFN-α/β and IL-6 at various time points after Ad inoculation. Activation of p38 and IRF3 was analyzed with phosphospecific antibodies, whereas nuclear translocation of NF-κB subunit p65 was used to analyze NF-κB activation. In MPI cells, the strong activation of p38, IRF3, and NF-κB was first detectable at 1 to 2 h p.i. and persisted for up to 8 h for NF-κB and at least up to 10 h for IRF3 and p38 (Fig. 2A). In contrast, in BMMs weak, delayed, and shorter activation of p38 was detected between 2 and 6 h and activation of IRF3 and NF-κB was not detected at all (Fig. 2A). Consistent with this, Ad-infected MPI cells exhibited early and strong production of type I IFN and IL-6, while only marginal production of these cytokines was observed at 16 h after the inoculation of BMMs (Fig. 2B). Murine AMs (Siglec F, F4/80, and CD11c triple-positive cells), which constitute the vast majority of freshly isolated bronchoalveolar lavage (BAL) cells (see Fig. S1A in the supplemental material), were also infected with Ad. Similar to the infection of MPI cells, and unlike that of BMMs, they exhibited early and strong phosphorylation of p38 and IRF3 (Fig. S1B) and a strong cytokine response (Fig. S1C).

10.1128/mBio.00670-17.1 FIG S1  AdGFP-induced innate immune activation of freshly isolated murine AMs. (A) AM gating strategy. Living immune cells were identified as 4',6-diamidino-2-phenylindole (DAPI)-negative, CD45 (pan-immune cell marker)-expressing BAL cells. AMs were further identified as Siglec F and F4/80 double-positive cells and constituted the large majority of immune cells (>95%). AMs strongly expressed CD11c but not CD11b. (B) Activation of p38 and IRF3 was analyzed in whole-cell lysates by Western blotting at the time points indicated. (C) IFN-α/β and IL-6 in cell-free supernatants were analyzed by ELISA at 16 h p.i. Download FIG S1, PDF file, 0.2 MB. Copyright © 2017 Maler et al. 2017 Maler et al. This content is distributed under the terms of the Creative Commons Attribution 4.0 International license.

In summary, these results document a large difference in the activation of Ad-stimulated innate signaling between MPI cells/AMs and BMMs, and this correlates with the efficiency of Ad transduction.

### Cytoplasmic sensing by cGAS mediates Ad-stimulated innate responses in MPI cells.

**FIG 3:**
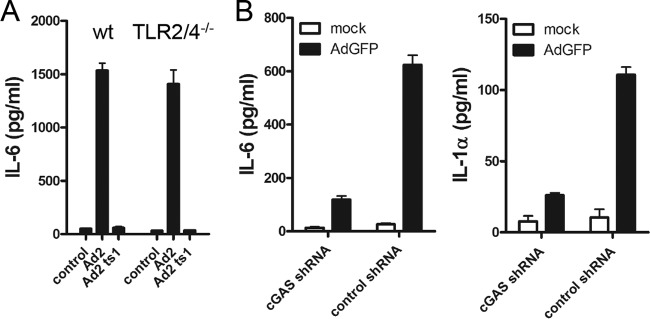
Ad-induced cytokine production in MPI cells is independent of TLR2 and TLR4 but dependent on cGAS. (A) IL-6 induction in WT and TLR2/TLR4^−/−^ MPI cells p.i. with WT Ad2 or mutant Ad2 ts1. (B) Cytokine induction in cGAS knockdown MPI cells inoculated with AdGFP or mock infected for 16 h.

The initiation of Ad-induced innate responses has been attributed to the cell surface receptors TLR2 and TLR4, endosomal TLR9, and more recently to the cytoplasmic DNA sensor cGAS (26, 27, 29). MPI cells deficient in both TLR2 and TLR4 showed no reduction of IL-6 production following Ad infection (Fig. 3A). Furthermore, infection of MPI cells with the Ad2 ts1 mutant, which is taken up into endosomes but does not enter the cytosol (33), did not induce detectable cytokine production (Fig. 3A), indicating that virus sensing at the plasma membrane or in the endosomes is not sufficient for induction of the innate responses in MPI cells. To test the role of cytoplasmic cGAS, we used short hairpin RNA (shRNA) to knock down this sensor in MPI cells. A C911 mismatch shRNA was used as a control (34). AdGFP transduction of cGAS knockdown cells resulted in significantly less cytokine production than in Ad-transduced MPI cells expressing the C911 control shRNA (Fig. 3B). In a control experiment, cytokine production in response to lipopolysaccharide (LPS) was not reduced in cGAS knockdown MPI cells (Fig. S2A). Notably, the knockdown of cGAS had no negative influence on the AdGFP transduction of MPI cells, as measured by GFP expression in infected MPI cells (Fig. S2B).

10.1128/mBio.00670-17.2 FIG S2  LPS-induced IL-6 production and Ad-induced GFP expression are independent of cGAS. (A) MPI cells were transduced with cGAS or control shRNA and stimulated with 100 ng/ml LPS. IL-6 was analyzed in cell-free supernatants at 16 h after stimulation. (B) GFP expression in MPI cells infected with AdGFP for 16 h after shRNA-mediated knockdown of cGAS (top, fluorescence microscopy; bottom, corresponding phase-contrast microscopy, Scale bars, 100 µm). Download FIG S2, PDF file, 0.4 MB. Copyright © 2017 Maler et al. 2017 Maler et al. This content is distributed under the terms of the Creative Commons Attribution 4.0 International license.

### Ad induces production of mature IL-1α in MPI cells but not in BMMs.

**FIG 4:**
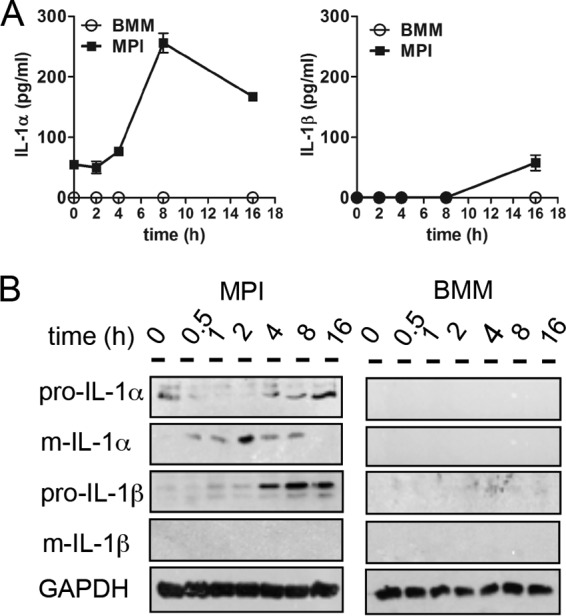
Ad-induced IL-1 production in MPI cells and BMMs. (A) Induction of IL-1α and IL-1β in cell-free supernatants of MPI cells and BMMs after Ad infection at the time points indicated. (B) Western blot analysis of MPI and BMM cell lysates at the time points indicated. GAPDH, glyceraldehyde-3-phosphate dehydrogenase.

We have previously demonstrated that LPS-stimulated MPI macrophages, unlike BMMs, secrete substantial amounts of IL-1α (31). Ad infection also induced a significant IL-1α response, which peaked 8 h after inoculation, and a marginal IL-1β response in MPI cells, but not in BMMs (Fig. 4A). We tested whether Ad infection also induces the maturation of these cytokines. For this purpose, we infected MPI cells and BMMs with Ad and analyzed the IL-1α and IL-1β forms in cell lysates by Western blotting at different times p.i. Ad infection resulted in the processing of preexisting immature IL-1α between 0.5 and 8 h p.i. and in the accumulation of pro-IL-1α starting after 4 h of stimulation (Fig. 4B). Furthermore, immature IL-1β, but not the mature IL-1β form, was induced in Ad-infected MPI cells at later time points (4 to 16 h p.i.) (Fig. 4B). Finally, no induction of IL-1α or IL-1β was detectable in lysates of Ad-infected BMMs.

### Blocking of the SR MARCO on MPI cells and AMs interferes with Ad entry and induction of cytokine production.

**FIG 5:**
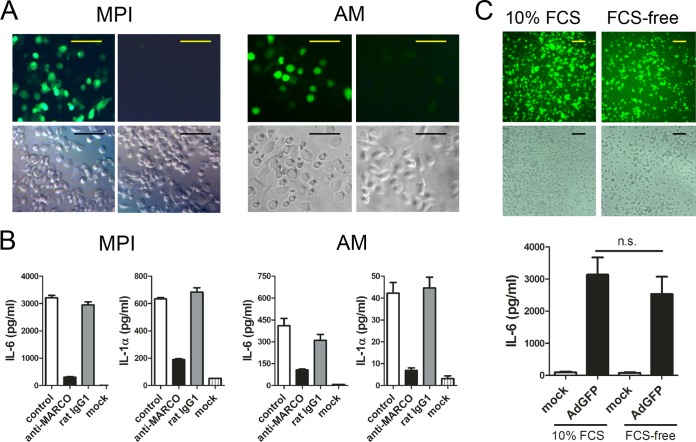
Ad transduction is strongly reduced by blocking MARCO but independent of serum factors. (A) GFP expression of cells infected with AdGFP in the presence of a MARCO-blocking (right) or isotype antibody (left). Scale bars, 100 µm. Top, fluorescence microscopy; bottom, phase-contrast microscopy. (B) Cytokine production of AdGFP-infected cells without antibody (control) and with a MARCO-blocking antibody or an isotype control. (C) GPF expression (top, fluorescence microscopy and corresponding phase-contrast microscopy) and IL-6 production (bottom) of AdGFP-infected MPI cells in the presence or absence of FCS. Scale bars, 100 µm. n.s., no statistically significant difference.

We investigated whether the differential expression of a ligand recognition receptor might be responsible for the greater sensitivity to Ad of MPI cells and AMs than of BMMs. As expected, the mRNA for CAR, the main receptor responsible for the uptake of species C Ads in most nonimmune cells, is not detectable in MPI cells and BMMs (Fig. S3A and B). Although the SRs SR-A and SREC1 have been implicated in Ad entry into macrophages (21–25), analysis of mRNA levels in MPI cells and BMMs did not show strong expression of these genes for these SRs in MPI cells (Fig. S3A and B), in agreement with our previously obtained microarray data (31). This suggested that these proteins are not involved in the differential sensitivity to Ad (Fig. S3). As shown in Fig. S3A to C and demonstrated previously (31, 35), another related microbial sensor, the SR MARCO, is highly expressed in MPI cells but absent from resting BMMs. SR-A is weakly expressed on both cell types (Fig. S3C). We also tested MARCO expression on various tissue-resident macrophages. We found no significant MARCO expression on liver and skin macrophages. However, peritoneal macrophages, AMs, and spleen marginal-zone macrophages exhibited substantial levels of MARCO on their surface (Fig. S4). These cell types have previously been reported to be very efficiently infected by Ads (36–39). We tested the possible involvement of MARCO in Ad transduction and in the elicitation of innate responses. For this purpose, we compared the GFP expression and IL-1α and IL-6 responses of AdGFP-infected MPI cells and AMs in the presence or absence of a monoclonal antibody that blocks ligand binding to MARCO (40). Preincubation for 30 min with an anti-MARCO antibody, but not with a control antibody, almost completely abolished both the AdGFP transduction and the virus-induced cytokine responses of these cell types (Fig. 5A and B). These results suggested a crucial role for MARCO in Ad sensing in MPI cells and AMs.

10.1128/mBio.00670-17.3 FIG S3  CAR, SREC1, SR-A, and MARCO expression in BMMs and MPI cells. (A) Comparison of mRNA expression (normalized intensity [NI] values) in BMMs and MPI cells by using microarray data published by Fejer et al. (G. Fejér, M. D. Wegner, I. Györy, I. Cohen, P. Engelhard, E. Voronov, T. Manke, Z. Ruzsics, L. Dölken, and O. P. da Costa, Proc Natl Acad Sci U S A 110:E2191--E2198, 2013, https://doi.org/10.1073/pnas.1302877110). (B) mRNA expression of the genes indicated validated by quantitative RT-PCR. Lung and B cell total RNAs were used as positive and negative controls, respectively. n.d., not detectable (C) FACS analysis of SR-A and MARCO expression in BMMs and MPI cells. Open histograms, specific antibody; gray-filled histograms, isotype control. Download FIG S3, PDF file, 0.2 MB. Copyright © 2017 Maler et al. 2017 Maler et al. This content is distributed under the terms of the Creative Commons Attribution 4.0 International license.

10.1128/mBio.00670-17.4 FIG S4  MARCO expression of different tissue macrophages. Macrophages were analyzed immediately after digestion (liver, skin, spleen) or lavage (peritoneum, lung) without prior enrichment by selective adhesion. Download FIG S4, PDF file, 0.2 MB. Copyright © 2017 Maler et al. 2017 Maler et al. This content is distributed under the terms of the Creative Commons Attribution 4.0 International license.

Several serum proteins have previously been shown to mediate Ad uptake by myeloid cells (20, 22); however, SRs mediate ligand internalization without their help (32). So far, the Ad transduction of macrophages in this study was carried out in the presence of fetal calf serum (FCS). As shown in Fig. 5C, the absence of FCS did not reduce the GFP expression (top) or the IL-6 response (bottom) of the infected cells. These results suggest that MARCO-expressing macrophages do not need opsonizing proteins to sense Ads.

### MARCO deficiency drastically reduces the Ad transduction rate and the reactivity of MPI cells and AMs to the virus.

**FIG 6:**
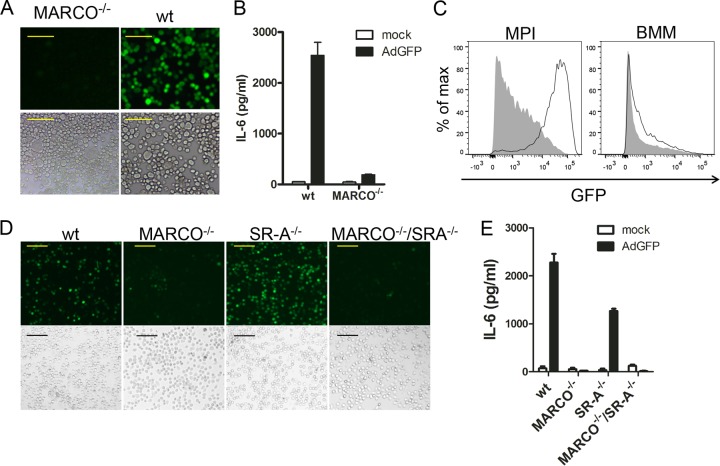
Effect of MARCO and SR-A deficiency on GFP expression and IL-6 production in macrophages infected for 18 h with AdGFP. (A) Fluorescence microscopy (top) and corresponding phase-contrast microscopy (bottom) of infected MARCO^−/−^ and WT MPI cells. Scale bars, 50 µm. (B) IL-6 concentrations in supernatants of mock-infected and infected MARCO^−/−^ and WT MPI cells. (C) FACS comparison of MARCO^−/−^ (gray-filled histogram) and WT (open histogram) MPI cells and BMMs at 16 h p.i. with AdGFP. (D) Fluorescence microscopy (top) and corresponding phase-contrast microscopy (bottom) of mock-infected and infected AMs from different knockout mice. Scale bars, 100 µm. (E) IL-6 concentrations in supernatants from mock-infected and infected AMs obtained from different knockout mice.

**FIG 7:**
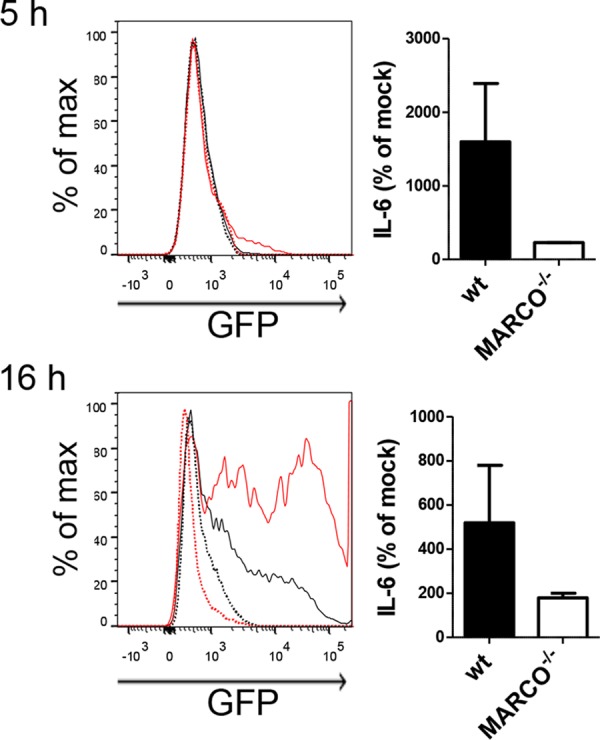
GFP expression and IL-6 response of peritoneal cells from naive WT and MARCO^−/−^ mice infected with AdGFP *in vitro* at 5 (top) and 16 (bottom) h p.i. Left, GFP expression in F4/80^+^ cells (histograms). Lines: black continuous, MARCO^−/−^ infected; black dotted, MARCO^−/−^ mock infected; red continuous, WT infected; red dotted, WT mock infected. Right, IL-6 levels in supernatants of peritoneal cells.

To test the importance of MARCO for Ad transduction, we generated an MPI macrophage line from MARCO^−/−^ mice. As expected, these cells expressed macrophage markers and responded to LPS (TLR4 ligand) and FSL-1 (TLR2 ligand) similarly to wild-type (WT) MPI cells (Fig. S5A and B). Importantly, however; compared to WT MPI macrophages, MARCO^−/−^ cells showed a strong reduction of Ad transduction (Fig. 6A and C) and Ad-stimulated cytokine responses (Fig. 6B), while the Ad infection of MARCO-deficient BMMs was not significantly reduced (Fig. 6C). A similar Ad-insensitive phenotype was also exhibited by two other MARCO^−/−^ MPI cell lines generated independently (not shown). In addition, we isolated AMs from WT, MARCO^−/−^, SR-A^−/−^, and MARCO^−/−^/SR-A^−/−^ doubly deficient mice and infected them with AdGFP. Compared to WT cells, MARCO^−/−^ and MARCO^−/−^/SR-A^−/−^ AMs showed strongly reduced GFP expression (Fig. 6D) and lacked an IL-6 response (Fig. 6E). However, the loss of SR-A alone resulted in no reduction of GFP expression and only a moderate decrease in the IL-6 response of infected AMs (Fig. 6E). As expected, the expression of macrophage surface markers and the LPS responses of WT and MARCO^−/−^ AMs were very similar (Fig. S5C and D). We also tested if MARCO expression contributes to Ad infection in peritoneal macrophages. We found that WT cells can be efficiently infected and activated (IL-6 response) by AdGFP; however, MARCO deficiency resulted in a marked reduction of AdGFP transduction and of the virus-triggered IL-6 response (Fig. 7). These results indicate that the absence of MARCO, but not SR-A, severely reduces Ad transduction and the innate responses in these macrophage types.

10.1128/mBio.00670-17.5 FIG S5  Surface protein expression and innate responsiveness of WT and MARCO^−/−^ MPI cells and AMs. (A) FACS analysis with anti-CD11b, -CD14, -MARCO, -Siglec F, -F4/80, and -CD11c antibodies. Open black histograms, WT cells; open red histograms, MARCO^−/−^ cells; gray-filled histograms, isotype control. (B) IL-6 production in cell-free supernatants of MPI cells at 16 h after stimulation with FSL-1 or LPS. (C) FACS analysis of BAL cells from naive WT and MARCO^−/−^ mice. AMs were identified as Siglec F and F4/80 double-positive cells as shown in Fig. S1A, and the AM number shown is the frequency of CD45 (pan-immune cell marker)-positive BAL cells. AMs were subjected to FACS analysis with anti-CD11b, -CD14, -Siglec F, -F4/80, and -CD11c antibodies. Open black histograms, WT cells; open red histograms, MARCO^−/−^ cells; gray-filled histograms, isotype control. (D) IL-6 production in cell-free supernatants of AMs at 16 h after stimulation with LPS at 100 ng/ml. Download FIG S5, PDF file, 0.3 MB. Copyright © 2017 Maler et al. 2017 Maler et al. This content is distributed under the terms of the Creative Commons Attribution 4.0 International license.

### Transfection of MARCO in macrophages leads to increased Ad sensitivity.

**FIG 8:**
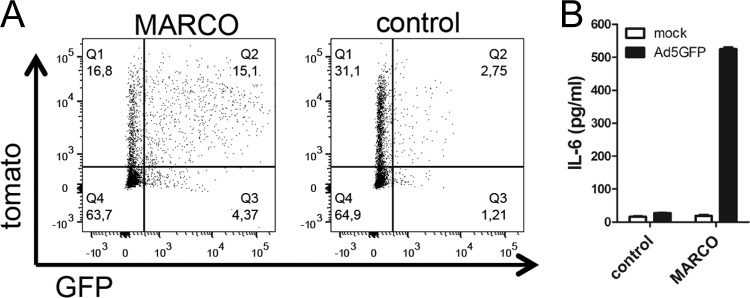
Expression of MARCO in RAW 264.7 macrophages increases the infection efficiency of and cytokine response to Ad. (A) Cells infected with AdGFP 16 h after transfection with murine MARCO or control plasmid DNA. Tomato was used as a reporter for successfully transfected cells. Cells were analyzed by FACS at 8 h p.i. (B) IL-6 was analyzed in cell-free supernatants 8 h after AdGFP infection.

To provide further evidence of the importance of MARCO, we transfected the murine RAW 264.7 macrophage line, which does not express MARCO (Fig. S6A, right), with a plasmid expressing full-length murine MARCO or with the same expression vector lacking MARCO. In both cases, expression of the cotransfected reporter tomato gene allowed discrimination between transfected (tomato-positive) and untransfected (tomato-negative) cells. As shown in Fig. 8A, both transfections yielded approximately 33% tomato-positive cells at the time of analysis. Transfection of RAW 264.7 cells with the MARCO-expressing plasmid resulted in MARCO surface expression only in tomato-positive cells (Fig. S6A, left). MARCO was absent from both tomato-positive and tomato-negative cells transfected with the control plasmid (Fig. S6A, left). MARCO- and control-transfected cells were infected with AdGFP and analyzed by flow cytometry for GFP and tomato expression at 8 h postinoculation. While 50% of the MARCO-transfected, tomato-positive cells expressed GFP, <10% of the control-transfected, tomato-positive cells expressed GFP, indicating a dramatic increase in Ad transduction due to the expression of MARCO. Pearson’s data analysis revealed a moderate positive correlation (*r* = 0.34; *P* < 0.0001) of GFP and tomato expression in MARCO-transfected RAW 264.7 cells, whereas no significant correlation (*r* = −0.02; *P* = 0.3361) was found in control-transfected cells (Fig. S6B). Furthermore, only cultures containing MARCO-transfected cells produced high levels of IL-6 in response to AdGFP (Fig. 8B).

10.1128/mBio.00670-17.6 FIG S6  Analysis of transfected RAW 264.7 cells. (A) FACS analysis of RAW 264.7 cells transfected with MARCO/tomato- or tomato-expressing plasmids. FACS analysis with anti-MARCO antibody was performed at 16 h after transfection. Left, frequencies of tomato^+^ cells; right, MARCO staining of tomato^+^ (top) and tomato^−^ (bottom) cell populations (open histogram, tomato-transfected cells; gray-filled histogram, MARCO/tomato-transfected cells). (B) Scatter dot plot of linear regression of AdGFP-infected MARCO- or control-transfected RAW 264.7 cells. Download FIG S6, PDF file, 0.5 MB. Copyright © 2017 Maler et al. 2017 Maler et al. This content is distributed under the terms of the Creative Commons Attribution 4.0 International license.

## DISCUSSION

In the present study, we show that compared to BMMs, Ad infects mouse AMs and AM-like MPI cells significantly more strongly and with accelerated kinetics. This higher infectivity rate explains why the Ad-induced proinflammatory cytokine responses of the latter two macrophage types are substantially stronger than those of BMMs. Previous studies indicated the possible involvement of cell surface TLR2 and TLR4, endosomal TLR9, and non-TLR cytosolic virus sensing in the Ad-stimulated innate cytokine responses (26–28). Using gene-deficient cells and the endosomal penetration-defective mutant Ad2 ts1, we excluded a significant involvement of TLR2, TLR4, and endosomal sensing in the Ad-triggered responses of MPI cells. Earlier, we showed that the IFN-α/β and IL-6 responses elicited by Ad strictly require viral endosomal escape (28). Here, we show that Ad transduction is much more efficient in MPI cells and AMs than in BMMs. This implies that both the delivery of viral particles to the cytosol and the nuclear import of viral DNA are efficient in these cells. Since incoming viral DNA is frequently and to a high level misdelivered to the cytosol (17), these results explain why the triggering of innate signaling, such as the activation of NF-κB, IRF-3, and p38, and the induction of cytokine responses are faster and stronger in AMs and MPI cells than in BMMs. The use of a different, less efficient Ad internalization route in BMMs may be responsible for the lack of detectability of NF-κB and IRF-3 activation upon infection and therefore for a very weak cytokine response. This is consistent with our previous results and the results of others showing that significantly higher Ad doses are necessary to elicit potent cytokine responses in BMMs (28, 41).

The finding that Ad ts1, a mutant unable to enter the cytosol, does not induce an IL-6 response in MPI cells confirms that Ad-triggered responses need cytosolic virus sensing. We show here that the knockdown of the cytosolic DNA sensor cGAS impairs the ability of Ads to activate MPI cells but does not affect adenoviral transduction measured by GFP transgene expression. These findings are in agreement with the major role of cytoplasmic virus sensing in MPI cells and with recent data showing that cGAS is a dominant Ad sensor for the induction of IFN-α/β but not for the nuclear translocation of viral DNA in the murine MS1 and RAW 264.7 cell lines (29).

A likely explanation for the considerable differences between the kinetics of transduction in AMs and MPI cells and those in BMMs was the differential expression of a specific cell surface receptor. The SR MARCO, which is highly expressed in alveolar, peritoneal, and MPI macrophages but not in BMMs (31), appeared to be a suitable candidate. Indeed, our data demonstrate that loss of MARCO function, achieved either by MARCO-blocking antibodies or by MARCO knockout in AMs and MPI macrophages, leads to severely reduced expression of Ad-transduced GFP and Ad-induced cytokine production. Moreover, the introduction of MARCO into RAW 264.7 macrophages, which do not express this protein, rendered the cells susceptible to Ad transduction and contributed to increased cytokine production. Thus, MARCO is decisively involved in the susceptibility of macrophages to Ad.

In the past, MARCO was shown to mediate the direct binding and internalization of various nonopsonized particles. Antibodies to the ligand-binding domain of this receptor can block the interaction (35). In agreement, our data obtained with MPI cells reveal that Ad infection and the elicitation of innate responses by Ad do not require the presence of serum but can be inhibited by the MARCO-blocking antibody. Therefore, it is likely that MARCO, similar to its role in the uptake of other microbial and nonmicrobial agents, mediates the internalization of Ad and thus promotes subsequent innate responses. This may occur in cooperation with other plasma membrane proteins. A cooperative action of CAR with integrins has been shown previously to initiate viral entry into epithelial cells (13). Tissue-resident macrophages are very heterogeneous (3), and variable expression of the factors responsible for Ad uptake and transduction, including MARCO, may explain the observed wide range of Ad infectibility of polyclonal MPI cells, AMs, and peritoneal macrophages.

SR-A, an SR related to MARCO and commonly expressed in various macrophage types, has been reported to be involved in the uptake of Ad by macrophages (21–25). Here, the comparison of GFP expression and cytokine production in AdGFP-infected AMs from WT, MARCO^−/−^, SR-A^−/−^, and MARCO^−/−^/SR-A^−/−^ mice indicated that this receptor, either alone or in combination with MARCO, does not play a major role in the Ad infection or activation of this macrophage type.

The identification of MARCO involvement in the susceptibility of cells to Ad agrees well with previous studies demonstrating that AMs and splenic marginal-zone macrophages, which strongly express MARCO, trap Ad particles very early p.i. (36, 39).

Ad-induced *in vivo* IL-1α production has been demonstrated previously in spleen. Di Paolo et al. showed that MARCO-positive spleen marginal-zone macrophages are a source of IL-1α in Ad-infected mice (36). They have also shown Ad-stimulated, IL-1α-mediated biological responses and the nuclear translocation of IL-1α. However, proteolytic maturation of this cytokine has not been demonstrated because similar to N-terminal pro-piece ppIL-1α, the immature IL-1α precursor can also readily translocate into the nucleus (42). As shown previously (31) and in the present study, MARCO-expressing AMs and MPI cells also exhibit a robust IL-1α response upon infection with Ads. We provide direct evidence of the intracellular processing of IL-1α in Ad-infected MPI cells. Both the full-length and processed IL-1α forms are biologically active, although the proinflammatory activity of the mature IL-1α protein is higher (43). Since Ads are important respiratory pathogens (44), and IL-1α has been shown to play a central role in lung pathologies induced by several microbial agents (45, 46), the elucidation of the mechanisms of Ad-induced IL-1α production in lung AMs is likely to have medical significance. Previously, di Paolo et al. (37) demonstrated that IL-1α produced by MARCO-expressing splenic marginal-zone macrophages is responsible for splenic neutrophil recruitment and the subsequent elimination of virus-infected cells. Thus, similar mechanisms may contribute to the AM-mediated control of respiratory Ad infection.

While MARCO is known to be involved in the innate sensing of various bacteria, much less is known about its role in virus infection and antiviral defense. MARCO was recently shown to play a role in the entry of herpes simplex virus and vaccinia viruses into keratinocytes, but macrophage infection and antiviral innate responses were not investigated (47, 48). MARCO was also shown to indirectly suppress early antiviral responses by removing cellular debris in the course of influenza virus infection (49). In contrast, our studies demonstrate a positive role for MARCO in the direct mediation of virus infection and triggering of antiviral responses in macrophages. Thus, MARCO may influence antiviral innate responses in a virus type-specific manner.

The expression of MARCO in specific subsets of naive tissue-resident macrophages can be important for the primary sensing of naturally occurring viral infections. AMs represent an early line of defense against respiratory pathogens; thus, the mechanisms shown here may critically influence the early stages of natural Ad infection. In addition, MARCO expression can be induced by inflammatory stimuli in macrophages normally not expressing this receptor (50). This may broaden the macrophage types contributing to the clearance of viral pathogens during the later phases of infection.

Our findings imply potential beneficial and harmful effects in medical applications using recombinant Ads. MARCO-mediated Ad infection of mononuclear phagocytes could contribute directly to efficient antigen processing and presentation. The cytokine responses induced by the vector could also provide adjuvant effects for vaccination. However, MARCO-mediated innate responses may be damaging, if the inflammatory response is too strong. Furthermore, certain MARCO-sensed pathogens, such as Ad and mycobacteria, induce hypersensitivity to LPS and other TLR ligands (51–53). Exposure of hypersensitive individuals to TLR-triggering pathogens may lead to uncontrolled inflammatory reactions and, in the worst case, to septic shock and death (54).

Our findings present a new target for further analyses of Ad-macrophage interactions. Future studies delineating the details of the Ad-MARCO interaction may provide tools to beneficially influence both naturally occurring Ad infections, as well as therapeutic approaches utilizing this viral vector.

## MATERIALS AND METHODS

### Mouse strains.

WT, MARCO^−/−^, SR-A^−/−^, and MARCO^−/−^/SR-A^−/−^ C57BL/6 mice (50) were bred under specific-pathogen-free conditions at the Max-Planck Institute in Freiburg or at the University Hospital of the Rheinisch-Westfälische Technische Hochschule in Aachen. Knockout mice strains were originally provided to S.G. by K. Tryggvason, Karolinska Institute, Stockholm, Sweden. Procedures were in accordance with institutional, state, and federal guidelines on animal welfare.

### Cell culture, Ad infection, and MARCO blocking.

Murine AMs were isolated by BAL, and BMMs and fetal liver-derived MPI cells were generated as previously described (31). Murine peritoneal cells were obtained by lavage with 5 ml of phosphate-buffered saline (PBS). Murine spleen cell suspensions were obtained by mechanical disintegration. To obtain skin cells, mouse ears were digested with Liberase Thermolysin Medium (TM; Roche) as described in reference 55. Minced mouse livers were digested with Liberase TM (Roche) for 30 min at 37°C, and Kupffer cells were further enriched by gradient centrifugation as described in reference 56. A549 adenocarcinoma-derived human alveolar basal epithelial cells and RAW 264.7 murine macrophages were cultured in Dulbecco’s modified Eagle’s medium (DMEM; Gibco) supplemented with 10% FCS (Biochrom), 100 µg/ml streptomycin (Gibco), 100 U/ml penicillin (Gibco), and 10 mM HEPES (Gibco). For the induction of cytokines, all cell types were used at a density of 5 × 10^5^/ml and plated on 96-well microtiter plates (Nunc) or 6-well plates (Nunc).

For Ad infection, replication-deficient, GFP-expressing species C human Ad5 (AdGFP), species C human WT Ad2, or mutant Ad2 ts1 was used. All viruses were used at a concentration of 1,000 particles/cell if not stated otherwise. GFP transduction was assessed by fluorescence and phase-contrast microscopy or fluorescence-activated cell sorter (FACS) analysis. The viruses were propagated and purified by CsCl_2_ density gradient ultracentrifugation as previously described (57). The concentration of virus particles was determined with a spectrophotometer. Optical density at 260 nm (OD_260_) − OD_330_ = 1 corresponds to 10^12^ virus particles/ml, and the ratio of viral particles to PFU was 20:1.

LPS from *Salmonella enterica* subsp. *enterica* serovar Minnesota R595 (Enzo Life Sciences) was used at a concentration of 100 ng/ml. MARCO ligand binding was blocked by incubation of cells with purified anti-mouse MARCO antibody (clone ED31; AbD Serotec) at a concentration of 20 ng/µl for 30 min prior to AdGFP infection. Purified rat anti-mouse IL-4 antibody (clone 11B11; BD Biosciences) was used as an isotype control.

### shRNA-mediated knockdown of cGAS in retrovirus-transduced MPI cells.

Plasmids pENTRpTER+ (430-1) (Addgene plasmid no. 17453) and pCQXIN X2 DEST (w310-1) (Addgene plasmid no. 17399) were generously provided by Eric Campeau, and a previously published protocol was followed (58). Viral RNA interference for mcGAS and its C911 control was established as previously described (34). The following oligonucleotides for mcGAS and its C911 control (adapted from reference 59) were designed with the Invitrogen shRNA Block-iT tool (Life Technologies, Inc.) and purchased from Microsynth AG, Balgach, Switzerland: mshcGAS_for, 5′ GATCCCGGATTGAGCTACAAGAATAGTGTGCTGTCCTATTCTTGTAGCTCAATCCTTTTTGGAAA 3′; mshcGAS_rev, 5′ AGCTTTTCCAAAAAGGATTGAG CTACAAGAATAGGACAGCACACTATTCTTGTAGCTCAATCCGG 3′; mshcGAS_ C911_for, 5′ GATCCCGGATTGAGGATCAAGAATAGTGTGCTGTCCTATTCTTGATCCTCAATCCTTTTTGGAAA 3′; mshcGAS_C911_rev, 5′ AGCTTTTCCAAAAAGGATTGA GGATCAAGAATAGGACAGCACACTATTCTTGATCCTCAATCCGG 3′.

shRNAs were cloned into entry vector pENTRpTER^+^ and recombined into Moloney murine leukemia virus destination vector pCQXIN X2 DEST by Gateway cloning with LR Clonase. Plasmids were validated by sequencing. Vesicular stomatitis virus G glycoprotein-pseudotyped murine leukemia virus retroviruses were generated in HEK 293T cells by the standard procedure and used to produce stably transduced MPI cells with G418 selection. The knockdown efficiency was 80%.

### FACS analysis.

Nonspecific binding was blocked by preincubation of cells with anti-CD16/CD32 antibody (clone 93; BioLegend) in 1% goat serum in PBS. MARCO was detected with purified mouse MARCO antibody (ED31; AbD Serotec) in combination with Alexa Fluor 647-conjugated goat anti-rat IgG (Molecular Probes). Fluorophore-conjugated CD11b (M1/70), CD14 (Sa14-2), CD11c (N418), F4/80 (BM8), and CD45 (30-F11) antibodies were from BioLegend; Siglec F (E50-2440) antibody was from BD Biosciences; and SignR1 (ERTR9) and SR-A (2F8) antibodies were from AbD Serotec. Data were acquired on a BD FACSCanto II cytometer and analyzed with FlowJo software (TreeStar).

### Western blotting.

Cell lysates were prepared in the presence of phosphatase inhibitor mixture II (Sigma), a protease inhibitor cocktail (Sigma), and phenylmethylsulfonyl fluoride (Sigma) with 100 µl of the respective lysis buffer per 10^6^ cells. Radioimmunoprecipitation assay buffer (20 mM Tris [pH 7.5], 150 mM NaCl, 1% NP-40, 0.5% deoxycholate, 1 mM EDTA, 0.1% SDS) was used to prepare whole-cell lysates, or the cells were fractionated for cytosolic and nuclear fractions as previously described (31). The quality of fractionation was checked with anti-glyceraldehyde-3-phosphate dehydrogenase (6C5; Acris Antibodies) and polyclonal rabbit anti-histone 3 (Merck Millipore) antibodies as described previously (31). Protein samples were blotted onto nitrocellulose membranes after denaturing SDS-polyacrylamide gel electrophoresis. Rabbit anti-NF-κB p65 (C-20; Santa Cruz Biotechnology), rabbit anti-phospho-IRF3 (Ser396) (4D4G; Cell Signaling Technology, Inc.), rabbit anti-phospho-p38 mitogen-activated protein kinase (Thr180/Tyr182) (12F8; Cell Signaling Technology, Inc.), polyclonal goat anti-IL-1α (R&D Systems), hamster anti-IL-1α (ALF-161; Santa Cruz Biotechnology), hamster anti-IL-1β (B122; R&D Systems) horseradish peroxidase-conjugated goat anti-mouse and goat anti-rabbit IgG (both Cell Signaling Technology, Inc.), mouse anti-hamster IgG (Santa Cruz Biotechnology), and rabbit anti-goat IgG (Jackson ImmunoResearch, Inc.) antibodies were used for immunoblotting. Blots were developed with the SuperSignal West Pico chemiluminescent substrate (Thermo Scientific).

### Cytokine detection.

IL-6, IL-1α, and IL-1β were quantified in cell-free culture supernatants by enzyme-linked immunosorbent assay (ELISA) with antibody pairs from BD Pharmingen (IL-6, IL-1β) and EBioscience (IL-1α) in accordance with the instructions of the manufacturer. IFN-α/β was measured with reference to a recombinant mouse IFN-β standard with an established luciferase expression-based bioassay as previously described (28).

### Transfection.

The mammalian expression vector pCmarcoiT was used to express the full-length murine MARCO protein. To construct the vector, full-length MARCO cDNA was amplified from MPI cell cDNA with forward primer 5′ GCCATGGGAAGTAAACAACTCC 3′ and reverse primer 5′ GTCAGGAGCATTCCACACCCGCA 3′ and cloned to give a cytomegalovirus-driven bicistronic transcript with the tandem-dimer tomato gene (referred to here as tomato) expressed from an internal translation initiation site. This allows identification by FACS and analysis of the transfected cells. RAW 264.7 cells were transfected with TurboFect Transfection Reagent (Thermo Scientific) in accordance with the manufacturer’s instructions.

### Quantitative RT-PCR.

RNA was prepared with Tri reagent (Sigma) in accordance with the manufacturer’s instructions. SuperScript II reverse transcriptase (Invitrogen) was used to reverse transcribe 2 µg of RNA. Real-time reverse transcription (RT)-PCR was performed with the LightCycler 480 SYBR green I master kit (Roche) and the Roche LightCycler 480 instrument with the following primers: Marco, forward primer 5′ ACCAGGCCTACCAGGTTTG 3′ and reverse primer 5′ ACCCTGCACTCCAGGTTTT 3′; Sra, forward primer 5′ CAGTCAGCATCCTCTTGTTCA 3′ and reverse primer 5′ GTCTTCTTTACCAGCAATGACAAA 3′; Srec1, forward primer 5′ GACTGGACCCGAAGGACA 3′ and reverse primer 5′ CGAGCCCAAGTTGGTGAG 3′; CAR, forward primer 5′ CCCTGGGGTTGCAAATAAG 3′ and reverse primer 5′ GATCCATCCACGAAGCATCT 3′; actin, forward primer 5′ GTCCACACCCGCCACCAGTTCG 3′ and reverse primer 5′ GGAATACAGCCCGGGGAGCATCGTC 3′. Data were normalized to β-actin expression and plotted on a relative scale for each gene by setting the cells with the highest level of expression to 100%.

### Data analysis, Pearson correlation, and statistics.

Data were analyzed with GraphPad Prism 5.0 software. Data are presented as mean values, and error bars show the standard errors of the means. Statistical significance was calculated with the unpaired *t* test (*, *P* < 0.05; **, *P* < 0.01; ***, *P* < 0.001). If not stated otherwise, representative results of at least three independent experiments are shown.
