# Erratum for Maler et al., “Key Role of the Scavenger Receptor MARCO in Mediating Adenovirus Infection and Subsequent Innate Responses of Macrophages”

**DOI:** 10.1128/mBio.01445-17

**Published:** 2017-09-26

**Authors:** Mareike D. Maler, Peter J. Nielsen, Nicole Stichling, Idan Cohen, Zsolt Ruzsics, Connor Wood, Peggy Engelhard, Maarit Suomalainen, Ildiko Gyory, Michael Huber, Joachim Müller-Quernheim, Wolfgang W. Schamel, Siamon Gordon, Thilo Jakob, Stefan F. Martin, Willi Jahnen-Dechent, Urs F. Greber, Marina A. Freudenberg, György Fejer

**Affiliations:** a Max-Planck-Institute of Immunobiology and Epigenetics, Freiburg, Germany; b Allergy Research Group, Department of Dermatology, Medical Center-University of Freiburg, Faculty of Medicine, University of Freiburg, Freiburg, Germany; c Faculty of Biology, University of Freiburg, Freiburg, Germany; d Institute for Virology, Medical Center-University of Freiburg, Faculty of Medicine, University of Freiburg, Freiburg, Germany; e Institute of Molecular Life Sciences, University of Zurich, Zurich, Switzerland; f School of Biomedical and Healthcare Sciences, Peninsula Schools of Medicine and Dentistry, University of Plymouth, Plymouth, United Kingdom; g Department of Pneumology, Medical Center-University of Freiburg, Faculty of Medicine, University of Freiburg, Freiburg, Germany; h Department of Molecular and Cell Biology, University of Leicester, Leicester, United Kingdom; i Institute of Biochemistry and Molecular Immunology, Medical Faculty, RWTH Aachen University, Aachen, Germany; j BIOSS Centre for Biological Signalling Studies, Faculty of Biology, and Center for Chronic Immunodeficiency (CCI), Medical Center-University of Freiburg, Faculty of Medicine, University of Freiburg, Freiburg, Germany; k Sir William Dunn School of Pathology, University of Oxford, Oxford, United Kingdom; l Department of Dermatology and Allergology, University Medical Center, Justus Liebig University Giessen, Giessen, Germany; m Biointerface Laboratory, Helmholtz Institute for Biomedical Engineering, Medical Faculty, RWTH Aachen University, Aachen, Germany

## ERRATUM

Volume 8, no. 4, e00670-17, 2017, https://doi.org/10.1128/mBio.00670-17. Affiliations i and l were given incorrectly. The affiliation line should appear as shown above.

