# Supplementary material for: Key Role of the Scavenger Receptor MARCO in Mediating Adenovirus Infection and Subsequent Innate Responses of Macrophages
Source: mBio. 2017 Aug 1;8(4):e00670-17. doi: 10.1128/mBio.00670-17 (PMC5539421; doi:10.1128/mBio.00670-17)
Supplement: FIG S1 [file mbo003173363sf1.pdf]

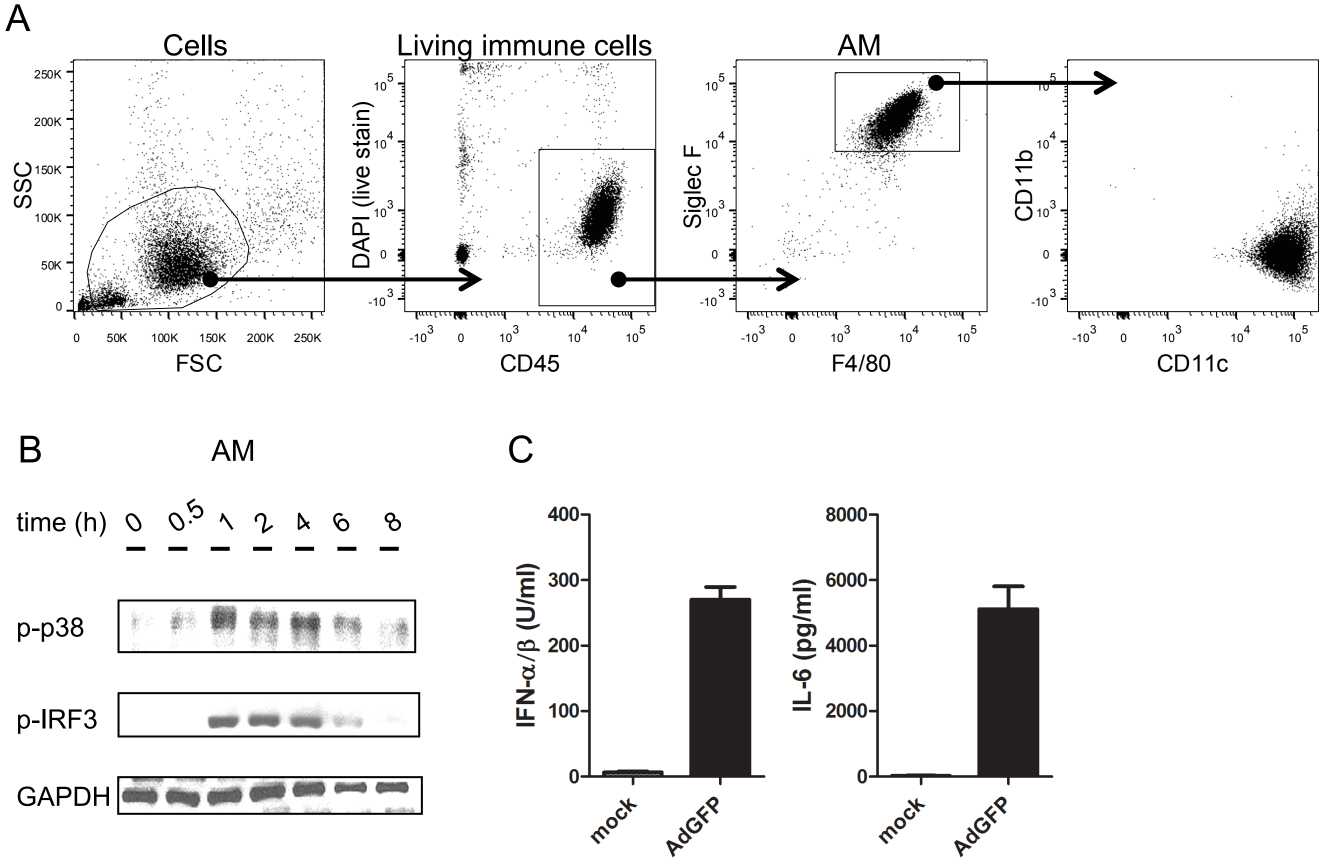

FIG S1. AdGFP-induced innate immune activation of freshly isolated murine AMs. (A) AM gating strategy. Living immune cells were identified as DAPI negative, CD45 (pan-immune cell marker) expressing BAL cells. AMs were further identified as Siglec F / F4/80 double positive cells and comprised the large majority of immune cells (> 95 %). AMs highly expressed CD11c but no CD11b. bottom: comparison of AM frequency before and after selective adhesion. (B) Activation of p38 and IRF3 was analyzed in whole-cell-lysates by western blot analysis at the indicated time points. (C) IFN-α/β and IL-6 were analyzed by ELISA in cell free supernatants 16 h post infection.
