# Supplementary material for: Key Role of the Scavenger Receptor MARCO in Mediating Adenovirus Infection and Subsequent Innate Responses of Macrophages
Source: mBio. 2017 Aug 1;8(4):e00670-17. doi: 10.1128/mBio.00670-17 (PMC5539421; doi:10.1128/mBio.00670-17)
Supplement: FIG S2 [file mbo003173363sf2.pdf]

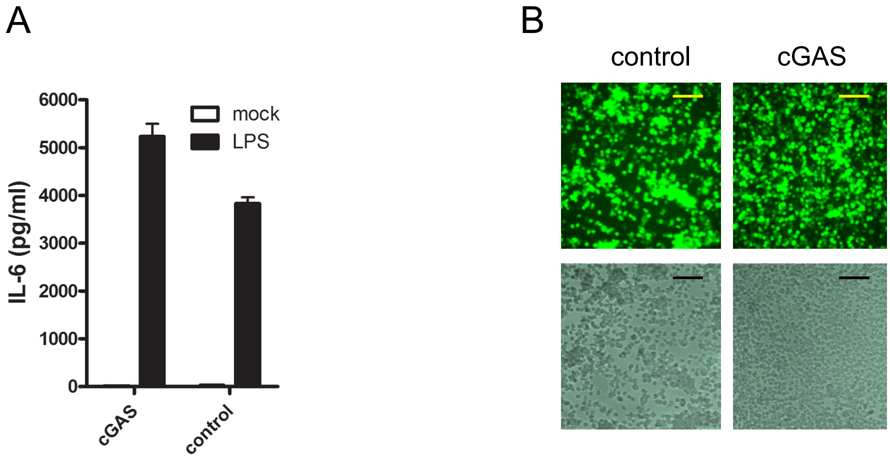

FIG S2. LPS-induced IL-6 production and Ad-induced GFP expression are independent of cGAS. (A) MPI cells were transduced with cGAS or control shRNA and stimulated with 100 ng/ml LPS. IL-6 was analyzed in cell free supernatants 16 h after stimulation. (B) GFP expression in MPI cells infected with AdGFP for 16 h after shRNA mediated knockdown of cGAS (top: fluorescence; bottom: corresponding phase contrast, Scale bars indicate 100 µm).
