# Supplementary material for: Key Role of the Scavenger Receptor MARCO in Mediating Adenovirus Infection and Subsequent Innate Responses of Macrophages
Source: mBio. 2017 Aug 1;8(4):e00670-17. doi: 10.1128/mBio.00670-17 (PMC5539421; doi:10.1128/mBio.00670-17)
Supplement: FIG S3 [file mbo003173363sf3.pdf]

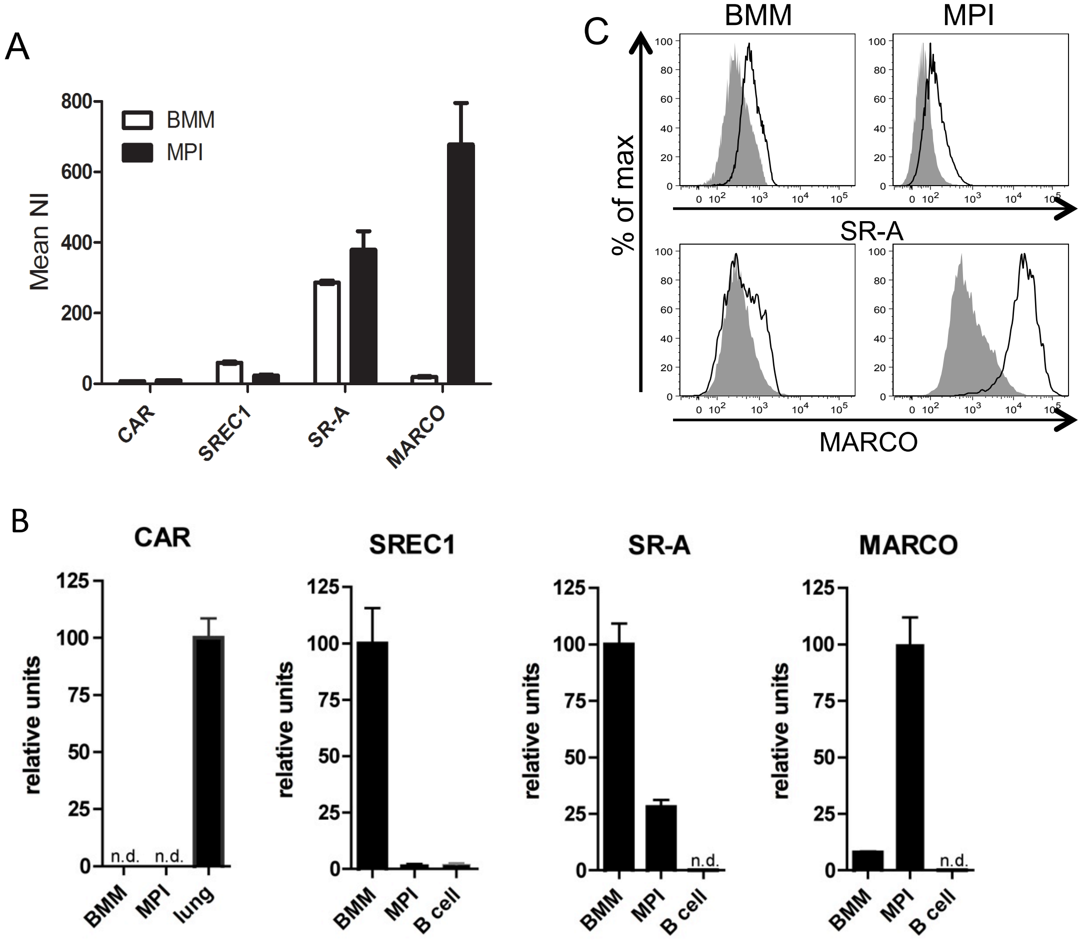

FIG S3. CAR, SREC1, SR-A and MARCO expression in BMM and MPI cells. (A) Comparison of mRNA expression (normalized intensity values; NI) in BMM and MPI cells using microarray data published in (1).

(B) mRNA expression of indicated genes validated by qRT-PCR. Lung and B cell total RNA was used as positive and negative control, respectively. n. d.: not detectable (C) FACS analysis of SR-A and MARCO expression in BMM and MPI cells . Open histograms: specific antibody, grey filled histograms: isotype control
