# Supplementary material for: Key Role of the Scavenger Receptor MARCO in Mediating Adenovirus Infection and Subsequent Innate Responses of Macrophages
Source: mBio. 2017 Aug 1;8(4):e00670-17. doi: 10.1128/mBio.00670-17 (PMC5539421; doi:10.1128/mBio.00670-17)
Supplement: FIG S4 [file mbo003173363sf4.pdf]

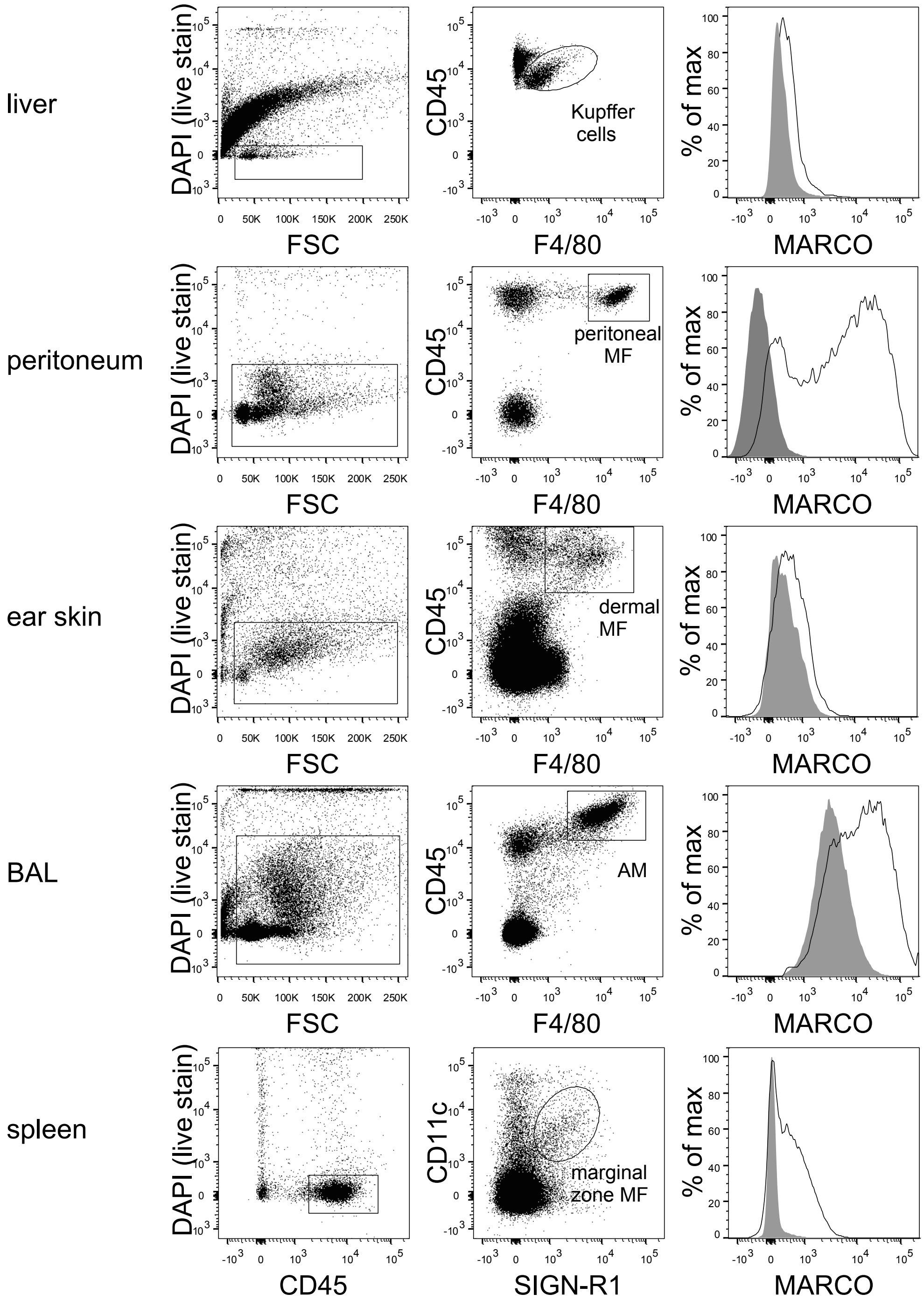

FIG S4. MARCO expression of different tissue macrophages. Macrophages were analyzed immediately after digestion (liver, skin, spleen) or lavage (peritoneum, lung) without prior enrichment by selective adhesion.
