# Supplementary material for: Key Role of the Scavenger Receptor MARCO in Mediating Adenovirus Infection and Subsequent Innate Responses of Macrophages
Source: mBio. 2017 Aug 1;8(4):e00670-17. doi: 10.1128/mBio.00670-17 (PMC5539421; doi:10.1128/mBio.00670-17)
Supplement: FIG S5 [file mbo003173363sf5.pdf]

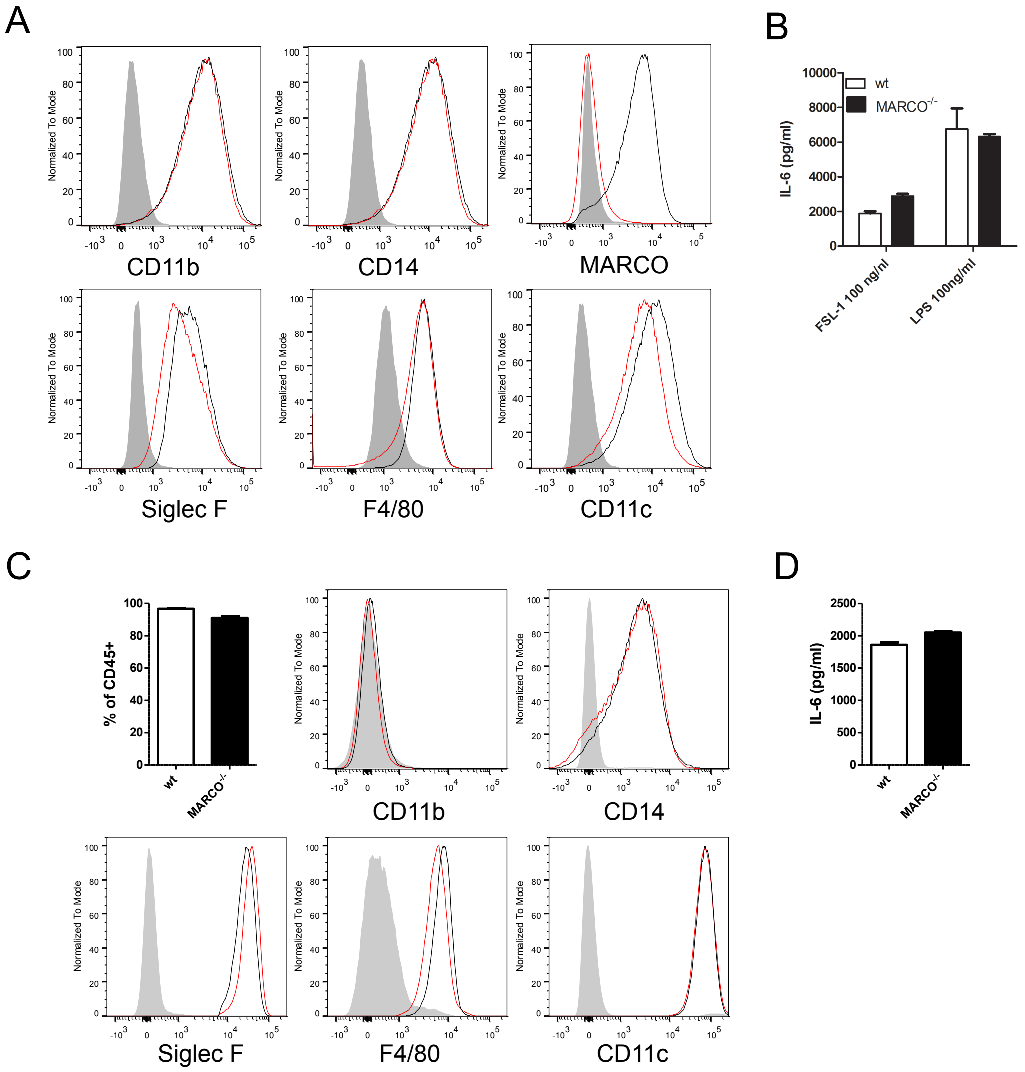

FIG S5. Surface protein expression and innate responsiveness of wt and MARCO$^{-/-}$ MPI cells and AM. (A) FACS analysis with anti-CD11b, -CD14, -MARCO, -Siglec F, -F4/80 and -CD11c. Open black histograms: wt cells, open red histograms: MARCO-/- cells, grey filled histograms: isotype control (B) IL-6 production in cell free supernatants of MPI cells 16 h after stimulation with FSL-1 or LPS. (C) FACS analysis of BAL cells from naïve wt and MARCO$^{-/-}$ mice. AMs were identified as Siglec F / F4/80 double positive cells as shown in Fig S1A and AM number is given as frequency of CD45 (pan-immune cell marker) positive BAL cells. AMs were FACS analyzed with anti-CD11b, -CD14, -Siglec F, -F4/80 and -CD11c. Open, black histograms: wt cells, open red histograms: MARCO$^{-/-}$ cells, grey filled histograms: isotype control (D) IL-6 production in cell free supernatants of AMs 16 h after stimulation with 100 ng/ml LPS.
