# Supplementary material for: Key Role of the Scavenger Receptor MARCO in Mediating Adenovirus Infection and Subsequent Innate Responses of Macrophages
Source: mBio. 2017 Aug 1;8(4):e00670-17. doi: 10.1128/mBio.00670-17 (PMC5539421; doi:10.1128/mBio.00670-17)
Supplement: FIG S6 [file mbo003173363sf6.pdf]

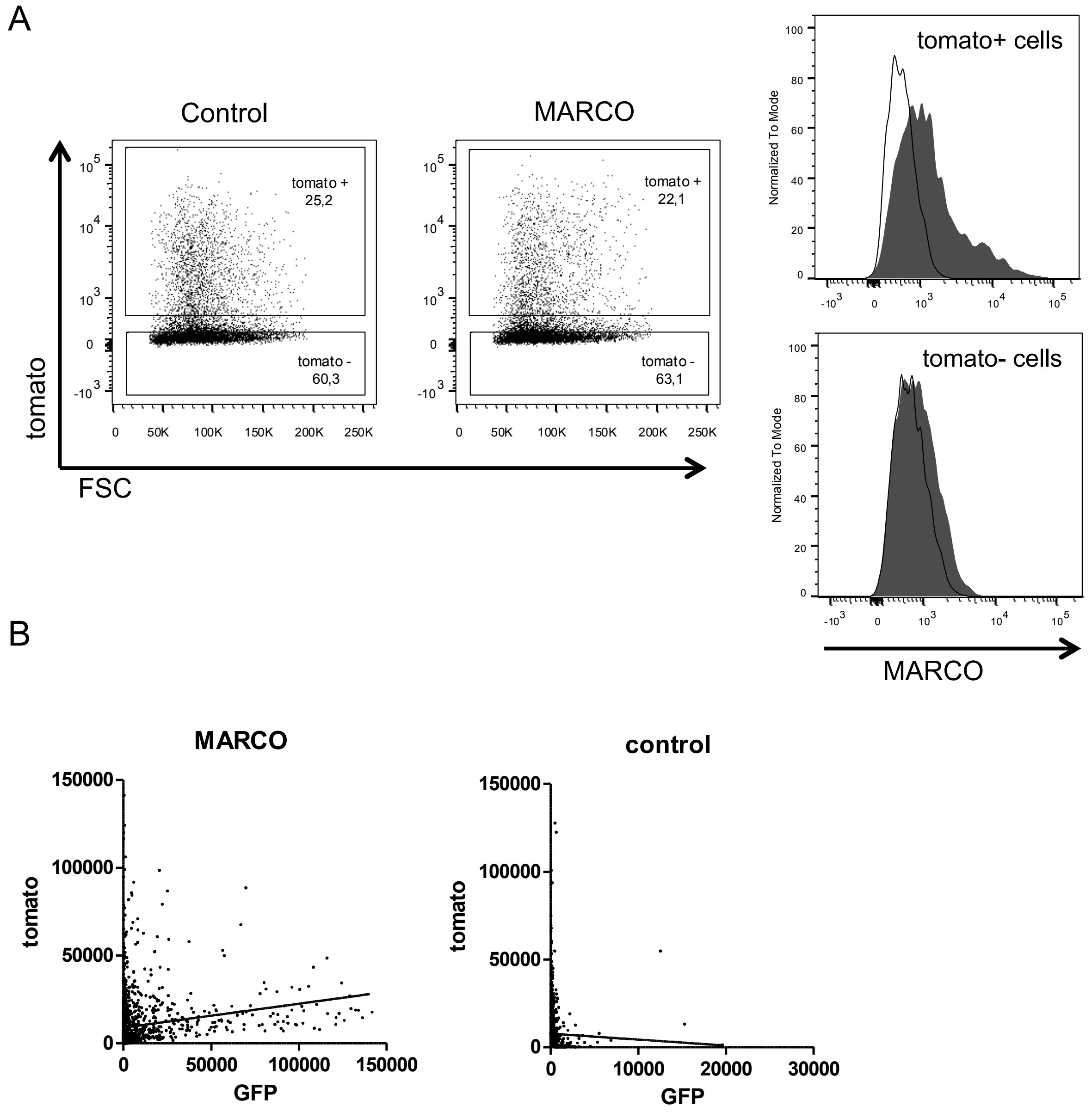

FIG S6. Analysis of transfected RAW264.7 cells. (A) FACS analysis of RAW264.7 cells transfected with MARCO/tomato- or tomato-expressing plasmids. FACS analysis with anti-MARCO was performed 16 h after transfection. Left: Frequencies of tomato+ cells. Right: MARCO staining on tomato+ (top) or tomato- (bottom) cell populations (open histogram: tomato-transfected cells, grey filled histogram: MARCO/tomato -transfected cells). (B) Scatter dot plot of linear regression of AdGFP infected MARCO or control transfected RAW264.7 cells.
